# Charcoal evidence that rising atmospheric oxygen terminated Early Jurassic ocean anoxia

**DOI:** 10.1038/ncomms15018

**Published:** 2017-05-12

**Authors:** Sarah J. Baker, Stephen P. Hesselbo, Timothy M. Lenton, Luís V. Duarte, Claire M. Belcher

**Affiliations:** 1 wildFIRE Lab, Hatherly Laboratories, University of Exeter, Prince of Wales Road, England, Exeter EX4 4PS, UK; 2 Deep time global change research group, Camborne School of Mines and Environment and Sustainability Institute, University of Exeter, Penryn Campus, Penryn, England, Cornwall TR10 9FE, UK; 3 Earth System Science Group, Laver Building, (Level 7), University of Exeter, North Park Road, England, Exeter EX4 4QE, UK; 4 MARE, Marine and Environmental Sciences Centre, Department of Earth Sciences, University of Coimbra, 3000-370 Coimbra, Portugal

## Abstract

The Toarcian Oceanic Anoxic Event (T-OAE) was characterized by a major disturbance to the global carbon(C)-cycle, and depleted oxygen in Earth's oceans resulting in marine mass extinction. Numerical models predict that increased organic carbon burial should drive a rise in atmospheric oxygen (*p*O_2_) leading to termination of an OAE after ∼1 Myr. Wildfire is highly responsive to changes in *p*O_2_ implying that fire-activity should vary across OAEs. Here we test this hypothesis by tracing variations in the abundance of fossil charcoal across the T-OAE. We report a sustained ∼800 kyr enhancement of fire-activity beginning ∼1 Myr after the onset of the T-OAE and peaking during its termination. This major enhancement of fire occurred across the timescale of predicted *p*O_2_ variations, and we argue this was primarily driven by increased *p*O_2_. Our study provides the first fossil-based evidence suggesting that fire-feedbacks to rising *p*O_2_ may have aided in terminating the T-OAE.

## Abstract (web summary)

Numerical models predict that during an oceanic anoxic event (OAE), increased organic carbon burial drives a rise in atmospheric oxygen leading to the event's termination. Here, the authors present evidence suggesting that the Toarcian OAE was terminated by rising oxygen and associated fire-feedbacks.

It has been suggested that projections of anthropogenic C-emissions likely exceed levels that have initiated Oceanic Anoxic Events (OAEs) in Earth's past^1^,^2^, implying that anthropogenic forcing of the Earth System may cause a future OAE, with implications for food security and for the oceans as a net C sink. OAEs are identified in the rock record by globally traceable organic-rich sediments and excursions within the δ^13^C record^3^,^4^, representing periods of major disturbance to the global C-cycle^4^. The study of OAEs therefore provides a geological test bed for understanding the functioning of global biogeochemical cycles under extreme conditions and rapid shifts in C-emissions.

Burial of organic carbon (C_(org)_) and reduced sulfur (pyrite) burial generate long-term build-up of oxygen to Earth's atmosphere^5^,^6^,^7^,^8^,^9^,^10^. It has thus been predicted that during an OAE, where increased burial of C_org_ and pyrite^11^ occur, that *p*O_*2*_ concentrations should have risen^12^ (Fig. 1). Wildfire responds strongly to *p*O_2_ (refs 13, 14, 15) and has been implicated in providing an essential long-term negative feedback, counteracting rising *p*O_2_ throughout geological time (for example, see refs 5, 12, 16). We therefore hypothesize that fire-activity should track projected *p*O_2_ changes across an OAE (Fig. 1).

Variation in the occurrence and abundance of charcoal throughout Earth history is taken to represent changes in fire-activity and appears to correlate with broad trends in the abundance of *p*O_2_ (refs 13, 17). A few studies have looked at the fossil-charcoal record within Devonian marine black shales and related them to changes in *p*O_2_ and fire-feedbacks^18^,^19^. However, fossil charcoal has not been studied across events such as OAEs in order to test the hypothesis that fire-feedbacks to *p*O_2_ may have acted together to rebalance the Earth system during these events^9^,^12^. Here we test this hypothesis by assessing the charcoal content of sediments from two sites deposited in the southern Laurasian Seaway, at Mochras in Wales, UK and Peniche, Portugal.

In this study, we find that charcoal abundances and therefore inferred wildfire-activity at both study locations are enhanced ∼1 Myr after the onset of the T-OAE. The enhancement of fire-activity is sustained for ∼800 kyr and peaks during the OAE termination. Variations in *p*O_2_ are anticipated to occur over Myr timescales^6^,^16^,^20^ due to the long residence time of oxygen within the ocean-atmosphere system^12^,^20^. The major enhancement of fire-activity occurring ∼1 Myr after the start of the T-OAE and lasting over ∼800 kyr is strikingly similar to the Myr timescale required for predicted global *p*O_2_ variations. Our findings therefore provide the first fossil-based evidence to support the postulation that OAEs are terminated by a rise in *p*O_2_ levels.

## Results

### Palaeo-wildfire

We find background charcoal abundances range between 22,000 and 75,000 particles per 10 g rock at Mochras, and 37,000 and 122,000 at Peniche prior to the T-OAE. Charcoal abundance rises briefly above background levels during the onset of the T-OAE, evidenced by 950,000 particles at Peniche (10.4 m log height) and 133,000 at Mochras (842 m depth), the start of which is widely identified in the rock record by a shift towards negative δ^13^C values at the culmination of a positive δ^13^C trend^3^ (Fig. 2). Charcoal abundances then decline to background values during the period of the negative carbon isotope excursion (CIE). Abundances remain at/below background levels until the final stages of the OAE (22.4 m at Peniche and 809 m at Mochras), where abundances increase to 293,000 particles at Peniche and 350,000 at Mochras. Although there appears to be variability within the charcoal abundance data, at both study locations abundances remain above background levels for ∼9.6 m at Peniche (between log heights 22.4 m and 32 m); and 23 m at Mochras (between core depths 809 m and 786 m), with the exception of one point at 803 m at Mochras, where abundances decline to 45,000 particles per 10 g of rock. Once the T-OAE has terminated, identified by the δ^13^C values returning to towards pre-excursion levels, abundances return to background levels as evidenced at Peniche from a mean of 411,000 particles between log heights 22.20 m and 32 m to a mean of 62,000 particles between 32 m and 38.2 m; and at Mochras from a mean charcoal abundance of 307,000 particles between core depths 809–786 m to a mean of 113,000 particles between depths of 785 m and 771 m.

### Palynofacies

The reported variations in charcoal abundance do not appear to be an artefact of preservation or changes in terrestrial organic delivery across the OAE, because variations in preserved phytoclasts (comprising terrestrial vegetal matter including charcoal, plant cuticles, pollen and spores and coalified material), show little correlation with the variations in charcoal abundance at either site (Fig. 2). This is further statistically supported by higher than 0.05 *P *values of *P*=0.384 for the Mochras section (where Pearsons *R* equalled −0.0186), and *P*=0.195 for Peniche (where *R* equalled −0.2153), indicating that the correlation between charcoal abundance and phytoclast abundance throughout the sections is not significant.

## Discussion

Fluctuations in the abundance of terrestrial phytoclasts have been used to indicate shifts in proximal palaeo-shoreline distance from the depositional site, as well as changes in preservation, for example, due to a switch from anoxic to an oxic environment^21^. At Mochras, the depositional site is anticipated to have gradually deepened, beginning at the OAE initiation and continuing throughout the *falciferum* ammonite zone into the *bifrons* ammonite zone beyond the studied section^22^. Interestingly, phytoclast abundances do not appear to reflect this deepening trend, suggesting that the site may have continued to experience a similar influx of terrestrial material throughout the OAE and beyond. Therefore this deepening is unlikely to explain enhanced charcoal abundance towards the end of the OAE; if anything deepening might be expected to decrease the amount of charcoal reaching the depositional environment at this site. Furthermore the lack of correspondence between variations in phytoclast abundance and charcoal abundance, implies that the enhanced number of fossil-charcoal particles that occur during the final stages of the OAE are unlikely to be due to a change in organic preservation, and therefore most likely reflect a real change in fire-activity. The charcoal is further unlikely to have been reworked up-section as there is no evidence of reworking in the biostratigraphic record of the sites, nor evidence for enhanced bioturbation at the onset of this phase of the OAE. Because charcoal abundance is limited below this point, reworking of the older sediments appears an unlikely source for the abundant charcoal that appears in the final phases of the OAE.

Published astronomically calibrated timescales for the Peniche section^23^,^24^, estimate a total duration of between ∼900 kyrs and ∼1.7 Myrs for the T-OAE, respectively. These timescales have previously been compared and correlated with Toarcian sediments exposed in Yorkshire, UK, which also illustrates a strong astronomical forcing within the δ^13^C_org_ record^25^. The section we have studied at Mochras provides twice the thickness and is considered to be stratigraphically more complete than that exposed in Yorkshire^26^,^27^. For the purpose of this study, we have used the most up-to-date published correlation of the Peniche and Mochras section,^28^ plotted alongside the most up-to-date published cyclostratigraphically calibrated timescale from the Peniche section^24^.

**Figure 1:**
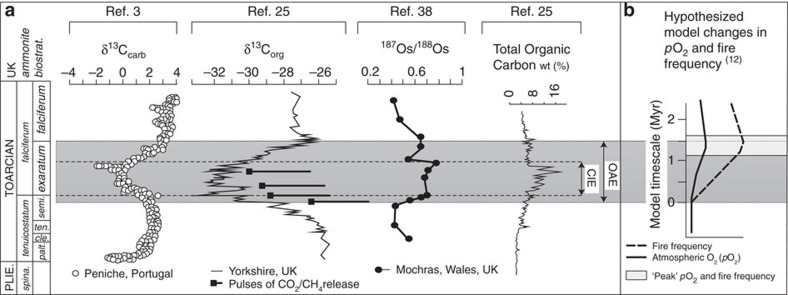
Summary of published data collected across the early Toarcian oceanic anoxic event. (**a**) Carbonate carbon isotope profile from Peniche and the organic carbon isotope profile from Yorkshire illustrating the step-wise nature of the negative excursion, and postulated pulses of light carbon release from Kemp *et al*.^25^ Plotted alongside are the osmium isotope profile from Mochras and total organic carbon content from the Yorkshire section. (**b**) Handoh and Lenton's^12^ hypothesized model changes in atmospheric oxygen and wildfire frequency across an oceanic anoxic event, with period of modelled peak oxygen and wildfire frequency highlighted.

**Figure 2:**
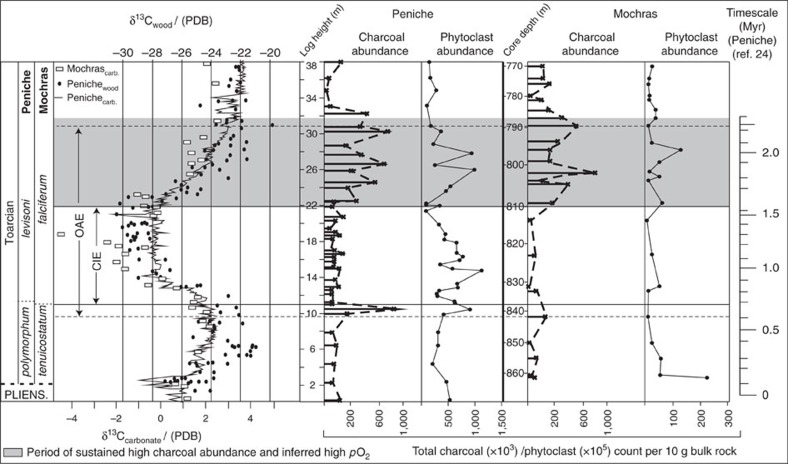
Charcoal and Phytoclast abundances across the T-OAE. Charcoal and phytoclast data collected in this study, plotted against published wood and carbonate carbon isotope profiles from Peniche and Mochras, and calculated cyclostratigraphic timescale from the Peniche section by ref. 24, 24. δ^13^C_wood_ and δ^13^C_carb_ profiles for Peniche are from ref. 3. The δ^13^C _carb_ for Mochras is from ref. 51 and correlated to the Peniche sequence using ref. 28.

Using the published timescale, we estimate that background charcoal abundances persisted for the duration of ∼600 kyrs (ref. 24) prior to the initiation of the OAE. The sustained increase in charcoal abundances at 22.4 m at Peniche and 809 m at Mochras, occurs at an estimated ∼1 Myrs after the OAE onset (Fig. 2), remaining elevated for an estimated 800 kyrs–1 Myrs before declining towards background abundances after the OAE termination. The major change in inferred wildfire-activity estimated at ∼1 Myrs after the OAE onset appears to corroborate the hypothesis of Handoh and Lenton^12^, suggesting that a rise in *p*O_2_ may have assisted in terminating the T-OAE by ventilating the ocean. However, while Handoh and Lenton^12^ predict that *p*O_2_ and thus fire-activity should be at a minimum at the start of the OAE and gradually rise throughout (Fig. 1), our analysis reveals a brief rise in fire-activity during the T-OAE initiation at both study sites, although albeit a much smaller rise in abundances at the Mochras site. Calculation of the *Z *scores for this brief rise in charcoal abundances (*Z*=10.43 at Peniche and *Z*=3.39 at Mochras) indicates that this increase in charcoal abundance at the onset, is statistically significant (larger than the critical *Z* value of 1.645) when compared to the background counts at both study sites. While, this observation is based on only one data-point in each section and requires corroboration, the brief rise in charcoal abundance occurs at the culmination of a positive δ^13^C trend. This could imply that the positive δ^13^C trend is indicative of an earlier prolonged increase in C_(org)_ burial, and therefore that *p*O_2_ began rising before the OAE.

Fire responses to rising *p*O_2_ are non-linear (see Fig. 4 in ref. 29); therefore, depending on the timing of the onset of C_(org)_ burial and the abundance of *p*O_2_ prior to the OAE, the fire responses could be variable throughout the OAE^29^. The *p*O_2_ estimates for 183 Ma range between 16 and 21% (ref. 17). If background *p*O_2_ were ∼19%, and the onset of C_(org)_ burial were capable of leading to a rapid 1% rise in *p*O_2_ by the start of the OAE, there would be a rapid rise in fire-activity as *p*O_2_ transitioned between 19 and 21% (ref. 29). Beyond 21% the fire response would slow but still continue to rise throughout the OAE with rising C_(org)_ burial and *p*O_2_, reaching a peak towards the end. However, most models estimate baseline *p*O_2_ at ∼20% (refs 16, 30), which would generate a slower fire response at the onset of enhanced C_(org)_ burial, with fire-activity gradually rising throughout and peaking at the end of the OAE. Therefore the initial peak in fire-activity would require a different explanation.

A study by McElwain *et al*.^31^ on a T-OAE section at Bornholm, Denmark, reveals that during the initiation, significant climatic changes occurred. Although, this sequence^31^ preserves only the lower part of the negative CIE, and was conducted at a higher resolution than that captured here, a rise in global temperatures and drying of the regional climate is inferred due to increased abundance of thermophilic plant taxa immediately before the first negative δ^13^C shift. Past Mesozoic global warming events have been shown to enhance fire activity^15^ and warm, dry periods are commonly linked with increased fire probability and large fire events^32^,^33^, which tend to be enhanced when dry periods succeed wetter periods that are favourable to fuel accumulation. Thus the brief enhancement of fire-activity may have been influenced by climatic changes occurring over timescales of a few hundred to thousand years close to the onset of the OAE; either driven directly by the climate change or from a resulting change in vegetation, the fuel for fires.

Following the brief rise in fire-activity, charcoal diminishes to near background levels, rather than rising gradually throughout the OAE. During the negative CIE, pulses of isotopically light C suggest enhanced input of CO_2_ into the atmosphere from volcanic sources^25^ leading to increased global temperatures^34^, sufficient to provoke methane (CH_4_)-hydrate dissociation^3^. This combined with an increase in terrestrial methanogenesis and a potential positive feedback associated with the decomposition of plant litter enabling further release of CO_2_ and CH_4_ from terrestrial sources (for example, refs 35, 36), thus created the large negative CIE and enhanced CH_4_ driven global warming. Coupled ocean-atmosphere models suggest an increase in global precipitation rates of +9 cm per year driven by the subsequent rises in CO_2_ (ref. 37). Increased continental weathering rates of up to ∼3 times larger than before the excursion^38^, have also been suggested based on a positive excursion in ^187^Os/^188^Os within the Jet Rock beds of Yorkshire and at Mochras^38^ (Fig. 1). These imply a warmer but wetter world; an expected feedback response to greenhouse induced warming^31^. Hence suppression of the rise in fire-activity throughout the negative CIE may be due to a significantly wetter climate, damping any *p*O_2_-fire response. Of significance however, is the ability of fire-activity to continue at background levels, which may indicate rising *p*O_2_ enabling fuels with higher moisture contents to continue to burn at a similar level to before the event^13^,^14^,^15^.

Towards the end of the T-OAE the δ^13^C record rises to more positive values again. At the same time, charcoal abundances at both sites increase, and remain elevated until the point of the T-OAE's termination. This synchronous rise begins at an estimated ∼1 Myr (ref. 24) after the start of anoxia, with charcoal abundances remaining elevated for an inferred ∼800 kyr (ref. 24), before returning to near background values (Fig. 2).

Following the negative CIE, palaeoclimatic conditions are hypothesized to have gradually cooled and dried, continuing well beyond the point of the T-OAE termination^34^,^39^. Climatic drying will have likely aided any *p*O_2_ driven enhancement of fire-activity, removing the suppression of fire under the wetter conditions of the CIE. Importantly however, beyond the OAE termination, charcoal abundances no longer track the inferred climate changes and instead decline despite the hypothesized climate continuing to dry and cool. Instead, after an inferred ∼800 kyr (ref. 24) of enhanced fire-activity the system appears to return to near background functioning, evidenced by the decline in charcoal at Peniche between 33.2m and 38.2 m, and at Mochras between 785m and 771 m.

Variations in *p*O_2_ are anticipated to occur over Myr timescales^6^,^16^,^20^, due to the long residence time of oxygen within the ocean-atmosphere system^12^,^20^, which is set by the large reservoir of oxygen in the atmosphere,∼3.7 × 10^19^ mol (ref. 20), divided by the relatively small flux of oxygen from C_(org)_ burial ∼18 × 10^12^ mol oxygen per year^1^,^20^ (and corresponding removal largely by oxidative weathering). The major enhancement of fire-activity for ∼800 kyr is strikingly similar to the Myr timescale required for hypothesized global *p*O_2_ reservoir variations^12^,^20^. In the model of Handoh and Lenton^12^, increased C_(org)_ burial across the OAE should lead to a gradual rise in *p*O_2_ and an increase in fire-activity, which leads to suppression of vegetation and a decline in chemical weathering rate (particularly of phosphorous) towards the end of the OAE (ref. 12). The latter prediction appears to be supported by the rapidly declining ^187^Os/^186^Os towards the end of the T-OAE (refs 38, 40). Some Earth system models (for example, COPSE (ref. 30)) depend on the sensitivity of fires to *p*O_2_ and the impact that fires have on vegetation biomass^16^,^30^ to regulate *p*O_2_. Fire is estimated to suppress Earth's present day vegetation biomass by 50% (ref. 41) at *p*O_2_ ∼21%, yet fire cannot be sustained (and therefore cannot suppress vegetation) below *p*O_2_ ∼15–17% (ref. 13). Even a modest increase in *p*O_2_ driven by enhanced C_(org)_ burial during the T-OAE, from, for example, the base level of *p*O_2_ (∼20%) estimated for the Jurassic^16^,^30^ to 21%, could have led to a 5% increase in burn probability due to the rapid response of fire to *p*O_2_ around this baseline level^13^. This in turn could have significantly enhanced the suppression of vegetation by fire^16^ by the end of the T-OAE. The resulting fire suppression of plant-driven phosphorus weathering may have assisted in terminating the T-OAE by reducing the input of phosphorus to the ocean and therefore productivity and oxygen demand in the water column. This effect would have combined with the direct effect of rising *p*O_2_ re-oxygenating the ocean and in turn causing phosphorus to be more efficiently removed to sediments^12^. Specifically, as the ocean starts to re-oxygenate this is predicted^12^ to increase the removal of phosphorus adsorbed to iron oxide minerals (Fe–P)^42^ and that preserved in organic matter (Org-P)^43^,^44^, lowering the ocean PO_4_ concentration and thus lowering oxygen demand in the water column—a strong positive feedback amplifying the re-oxygenation of the ocean. The data presented here provides the first fossil evidence that *p*O_2_ driven fire-feedbacks may have played a significant role in terminating ocean anoxia. We note that further work at additional study site(s) away from the Tethys region would be required to provide evidence that this fire response to rising *p*O_2_ was global in extent.

In conclusion, the observed increase in abundance of fossil charcoal, taken as a proxy for fire-activity occurring towards the end of the T-OAE is hypothesized to be primarily driven by increased *p*O_2_, providing the first fossil-based evidence to support the postulation that OAEs are terminated by a rise in *p*O_2_ levels. Thus the response of fire to Earth system perturbations across the T-OAE appears to capture a geologically rapid enhancement of *p*O_2_ implying that relatively small but significant changes in this key atmospheric gas may be possible over the timescale of an OAE. Such rapid C-cycle driven changes to *p*O_2_ suggest that new higher time-resolution models of *p*O_2_ over Earth's history may be required to explore the relationship between changes in C-cycling and Earth system functioning. This is critical because it appears that oxygen-fire feedbacks have the ability to regulate key processes that help re-oxygenate the ocean during perturbations to C-cycling and return the Earth system to background functioning. Given that the modern ocean is ‘on the edge of anoxia'^12^ and observations that the Earth system may take millions of years to regain background function if the ocean is tipped into an anoxic state, it will be critical to manage anthropogenic disruption to the C-cycle and maintain the natural functioning of wildfire-activity in order to regulate the Earth system within habitable bounds.

## Methods

### Study locations

**Figure 3:**
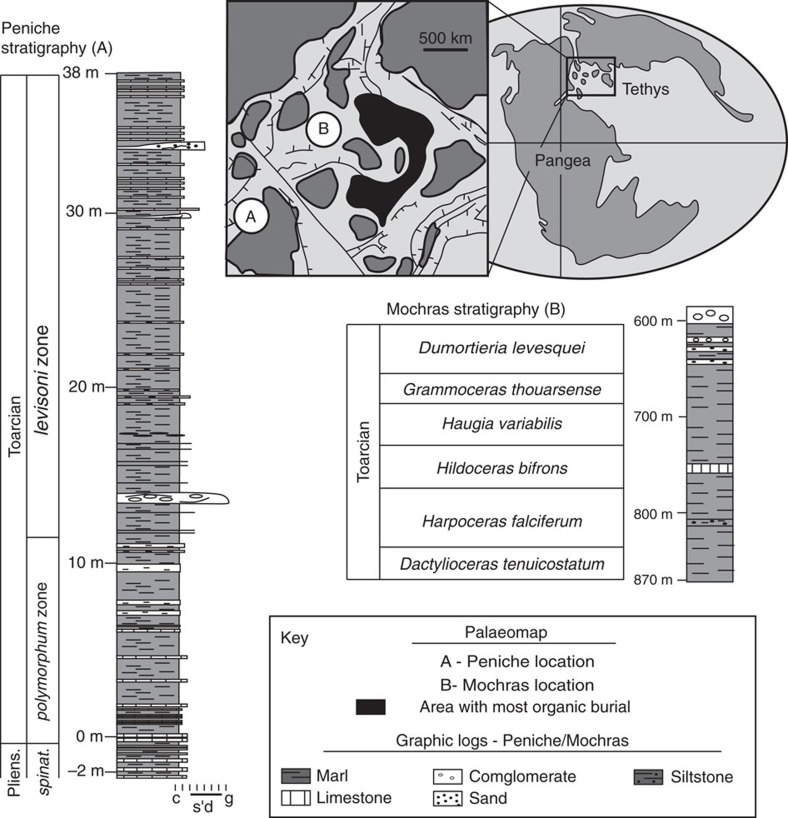
Jurassic Palaeo-map of the sample localities and relative site stratigraphies for the Mochras and Peniche sections. Palaeo-map and Peniche log were edited from ref. 3. Mochras log edited from ref. 26

Two sites were studied that record the T-OAE, deposited within the southern Laurasian Seaway; Peniche in Portugal and Mochras in the UK (Fig. 3). At Peniche, the Pliensbachian-Toarcian carbonate ramp succession is particularly well exposed, including the Toarcian GSSP (for example, see ref. 45). The deepest part of the ramp (∼200 m), represented by the Praia do Abalo sample locality, was bounded by the high-relief Berlenga–Farilhões horst, which provided terrestrial material to the sample site^46^. The site is unlikely to be influenced by a rise in sea level, as the horst would not have become more distal from the depositional area^3^. In Wales, UK, the Llanbedr (Mochras Farm) core, referred to as Mochras, drilled in 1967–1969, provides a complete section of mostly Early Jurassic sediments dating from the Late Rhaetian to Late Toarcian^47^,^48^. The sediments were deposited within a basinal marine setting, which became deeper during the initiation of the OAE, continuing throughout the *falciferum* zone into the *bifrons* zone (beyond the studied section)^22^ influenced by nearby terrigenous sources around the Cardigan Bay area^48^. Both sites are within the Laurasian Seaway (see Hesselbo and Pieńkowski^49^ and references therein), and are anticipated to capture regional signals of burning from the nearby emergent land.

### Sample collection and processing

**Figure 4:**
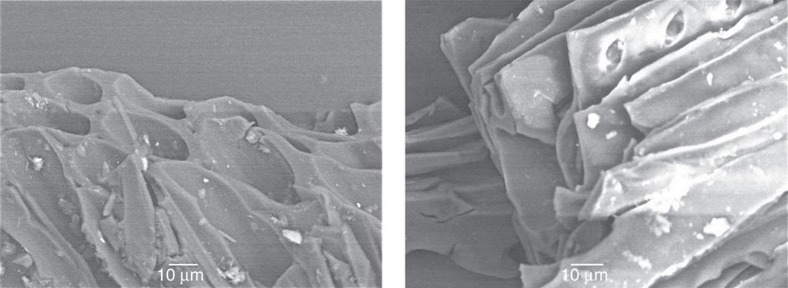
SEM images of charcoal fragments from the Peniche section. The fragments show the preservation of cellular anatomy and fused/homogenized cell walls—a key feature in charcoal identification.

Rock samples were collected from the exposed cliff sections in Peniche, and from the Mochras core, stored at British Geological Survey, Keyworth. The samples were picked from marl units, characterized by minor lithological changes and are considered to have been deposited within relatively uniform palaeoenvironments, minimizing the distortion of any fire-signals observed. Fossil charcoal was extracted from the Peniche and Mochras rock samples (48 samples in total were processed and analysed) using standard palynological acid maceration techniques. The remaining organic particles were sieved using a 125 μm mesh, where both size fractions were collected. The >125 μm size fractions were analysed using a binocular microscope, where all charcoal particles in each 20 g sample were quantified. The <125 μm fraction retained was quantified by evenly dispersing the organic particles in a known quantity of water. A known volume was then pipetted and made into slides using standard palynological techniques. Two transects of each slide were quantified, and scaled up to the known quantity of the <125 μm sample^50^. Selected particles were studied using a scanning electron microscope (SEM) to confirm their identification as charcoal (Fig. 4). To ensure changes in fossil charcoal concentrations were not biased by a change in nature or abundance of terrestrial organic material, a palynofacies analysis of each sample was conducted, quantifying the abundance of pollen and spores; plant cuticle, amorphous organic matter and coalified particles (Supplementary Data 1).

### Data availability

All data generated and analysed in this study are included in this published article and its supplementary information file.

## Additional information

**How to cite this article:** Baker, S. J. *et al*. Charcoal evidence that rising atmospheric oxygen terminated Early Jurassic ocean anoxia. *Nat. Commun.* **8,** 15018 doi: 10.1038/ncomms15018 (2017).

**Publisher's note:** Springer Nature remains neutral with regard to jurisdictional claims in published maps and institutional affiliations.

## Supplementary Material

Supplementary Data 1 Summary of raw data collected
